# Probing Real Sensory Worlds of Receivers with Unsupervised Clustering

**DOI:** 10.1371/journal.pone.0037354

**Published:** 2012-06-06

**Authors:** Michael Pfeiffer, Manfred Hartbauer, Alexander B. Lang, Wolfgang Maass, Heinrich Römer

**Affiliations:** 1 Institute for Theoretical Computer Science, TU Graz, Graz, Austria; 2 Institute of Neuroinformatics, University of Zurich and ETH Zurich, Zurich, Switzerland; 3 Institute for Zoology, Karl Franzens University, Graz, Austria; Heidelberg University, Germany

## Abstract

The task of an organism to extract information about the external environment from sensory signals is based entirely on the analysis of ongoing afferent spike activity provided by the sense organs. We investigate the processing of auditory stimuli by an acoustic interneuron of insects. In contrast to most previous work we do this by using stimuli and neurophysiological recordings directly in the nocturnal tropical rainforest, where the insect communicates. Different from typical recordings in sound proof laboratories, strong environmental noise from multiple sound sources interferes with the perception of acoustic signals in these realistic scenarios. We apply a recently developed unsupervised machine learning algorithm based on probabilistic inference to find frequently occurring firing patterns in the response of the acoustic interneuron. We can thus ask how much information the central nervous system of the receiver can extract from bursts without ever being told which type and which variants of bursts are characteristic for particular stimuli. Our results show that the reliability of burst coding in the time domain is so high that identical stimuli lead to extremely similar spike pattern responses, even for different preparations on different dates, and even if one of the preparations is recorded outdoors and the other one in the sound proof lab. Simultaneous recordings in two preparations exposed to the same acoustic environment reveal that characteristics of burst patterns are largely preserved among individuals of the same species. Our study shows that burst coding can provide a reliable mechanism for acoustic insects to classify and discriminate signals under very noisy real-world conditions. This gives new insights into the neural mechanisms potentially used by bushcrickets to discriminate conspecific songs from sounds of predators in similar carrier frequency bands.

## Introduction

In order to fulfill its task of shaping the behavior of organisms, the sensory system and the brain have to rely on information about the “outside” physical world, provided by the sense organs, which respond to different forms of energy. The information is transmitted via afferent nerves and encoded in trains of action potentials. The brain, by decoding this information, has to make adaptive assumptions about what had happened in the physical world. A central issue in sensory physiology deals with the coding and decoding mechanism(s) in the sense organs and central nervous system, respectively. Whereas early work concentrated on information provided by the average spike count over an appropriate time window (or firing rate in action potentials/second), it soon became clear that codes using the precise timing of action potentials would make more efficient use of the capacity of afferent lines to the brain. Yet, the mechanisms by which stimuli are represented in the timing of spikes are still not fully understood [1]–[3].

Irrespective of the sensory system investigated, recordings of single sensory neurons, or first-order sensory interneurons, always reveal isolated spikes and spikes grouped as bursts, i.e. short episodes of high-frequency action potential firing (e.g. [4]–[6]). These bursts - in contrast to single spikes-have been suggested to have particular importance for the function of the brain (review [7]), and in sensory systems bursts convey the important stimulus features [6], [8]. Yet, the problem of extraction of characteristic features within these bursts for identifying stimulus features and for object classification is difficult because spike trains exhibit variability. For insects and the acoustic modality, [9] reviewed the sources for such variability, and how it affects the processing of temporal patterns of acoustic signals. For example, one important source for such variability in the auditory modality results from the fact that in real world situations individuals are exposed to multiple sound sources, originating from different locations, or that signals are degraded and attenuated on the transmission channel between sender and receiver [10]–[14]. Internal noise as a result of stochastic processes within the nervous system is a further source for spike train variability.

As a result of the unavoidable noisiness of spike trains in neurons of sensory pathways one should expect the evolution of mechanisms in the nervous system leading to a reduction of the effects caused by false stimulus feature extraction and/or classification due to noise. On the other hand, minute variations in spike trains may well reflect differences between objects or object classes which are important for the receiver, such as small differences in the size of a sender, or the loudness or frequency composition in the sound signal of a mate. Such small differences, in contrast to those caused by noise, should be preserved during sensory processing, since they represent the neuronal basis for discrimination between mates or other decisions of importance for the receiver.

If bursts of action potentials contain the information about relevant features of objects or object classes, it should be possible to unambiguously distinguish 1) bursts of spikes elicited in response to a given stimulus from those bursts which resulted from noise, and 2) from bursts elicited in response to stimuli with different features. Various attempts have been made in the past to identify algorithms for such a task.

In this paper we present a set of machine learning tools to analyze and discriminate burst data while preserving most of the information about precise firing times, which is crucial within the auditory system. Our approach combines the Victor-Purpura spike metric [15]–[17] and the recently developed affinity propagation algorithm [18], a non-parametric clustering algorithm based on principles from probabilistic inference. Affinity propagation has lead to excellent results for a number of large datasets, and our study presents its first application to the discovery of burst patterns. This allows us to find meaningful spike patterns also in the responses to environmental noise signals, which may carry information about the identity or location of different sound sources.

We present data from a model system using an identified neuron approach in an acoustically communicating insect. This system offers several advantages for studying sensory burst coding over previous ones: 1) All recordings stem from the same identified neuron (called omega-neuron; [19]) in different preparations. 2) The first-order neuron in the auditory pathway integrates sensory information from a very limited number of receptor cells in the ear (20–40 receptors). 3) Recordings can be obtained for several hours, and most importantly, 4) portable preparations have been developed to make recordings directly in the insects’ natural environment, such as the tropical rainforest [13], [20], [21]. This permits to study sensory coding under the most natural conditions possible. Broadcasting well defined acoustic stimuli from some distance to the preparation, while recording the response of the neuron to these stimuli and to the background noise allows us to gain new insights about the characteristics and reliability of burst coding.

## Results

### The Omega Neuron and Experimental Setup

**Figure 1:**
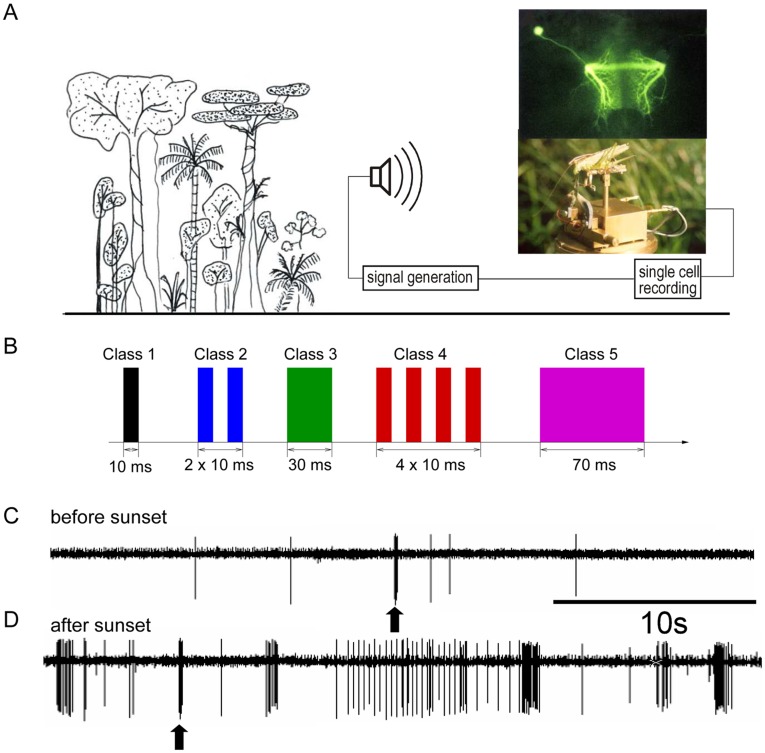
Experimental arrangement for long-term recordings of single cell activity in the tropical rainforest. **A**) Illustration of the experimental arrangement. The inset shows the morphology of the cell within the prothoracic ganglion after intracellular dye injection (upper part), and a prototype of the portable preparation. **B**) Illustration of the five stimulus classes played to bushcrickets during experiments. 1) Single pulse of 10 ms; 2) double pulse with 10 ms duration each, separated by an interval of 10 ms; 3) 30 ms pulse; 4) four repetitive pulses, 10 ms each, separated by an interval of 10 ms; 5) 70 ms pulse. **C, D**) Examples of recordings made at about one hour before sunset (**C**), and 45 minutes after sunset (**D**), when the background noise level had increased from 40 dB SPL to 65 dB SPL. Note that in the low noise situation only a stimulus (arrow) elicited a short burst of spikes, whereas after sunset the neuron fires many bursts also in response to the acoustic background.

A total of 27 adult male and female bushcrickets (*Docidocercus gigliotosi*) were used for this study. We recorded the activity of an identified auditory interneuron, the so-called omega neuron, in the field, using a technique introduced in [20] and [22], and explained in more detail in the Materials and Methods section. The morphology of the cell, as revealed from intracellular dye injection, is shown in Figure 1A (inset). It is a local neuron in the prothoracic ganglion and receives excitatory input from almost all of the 20–40 receptors in the hearing organ [23]. The tuning of the cell reflects the broad-band hearing sensitivity of the insect, matching both the frequencies of the conspecific calling song, and ultrasonic frequencies up to 100 kHz, thus including bat echolocation calls as well. As in other bushcricket species, the sensitivity of auditory receptors in *D. gigliotosi* differs by only a few dB from the sensitivity of the omega cell at most frequencies except below 5 kHz [24]. Furthermore, in response to a stimulus above its threshold, the neuron fires bursts of action potentials and copies the temporal pattern of an acoustic stimulus in a tonic manner. Altogether these attributes make outdoor recordings of the activity of the omega cell very suitable for studying sensory coding under realistic, i.e. outdoor conditions in the animals’ own natural habitat.

The study was conducted between 2002 and 2004 on Barro Colorado Island, located in central Panama within Gatún Lake, part of the Panama Canal. *D. gigliotosi* is a tropical insect living predominantly in the rainforest understorey, and all its activity, including acoustic communication, is restricted to the night. Thus, all recordings were made during times after sunset (about 6 p.m. local time) except for control measurements (see Materials and Methods).

During some of the recording sessions, five different stimuli at an intensity 20 dB above the threshold of the preparation were broadcast through a speaker. The stimuli differed in duration and the number of pulses (see Figure 1B), and were broadcasted every 10 seconds, which is within the range of the naturally occurring intervals in the calling song of this insect [21]. Males produce single or double syllables, repeated typically once every 10 seconds. Thus, the stimulus classes 1,2, and 3 in Figure 1B can be seen as representative for the variation of conspecific signals. Classes 4 and 5 are artificial stimuli that were used for control, and never occur in this species.

### Burst Coding of Acoustic Signals in Natural Habitats

A typical result for the effect of background noise on sensory coding is shown in Figure 1. The receiver was placed within the rainforest at 17.00 hrs 2 m from a speaker broadcasting a single sound pulse of 10 ms, at a sound pressure level of 20 dB above the threshold of the cell (which corresponds to intermediate distances of 5–10 m between sender and receiver). Since a female has no a priori knowledge about the presence of a male signal, her only information about a signal is encoded in afferent nervous activity such as the one shown in the upper recording.

**Figure 2:**
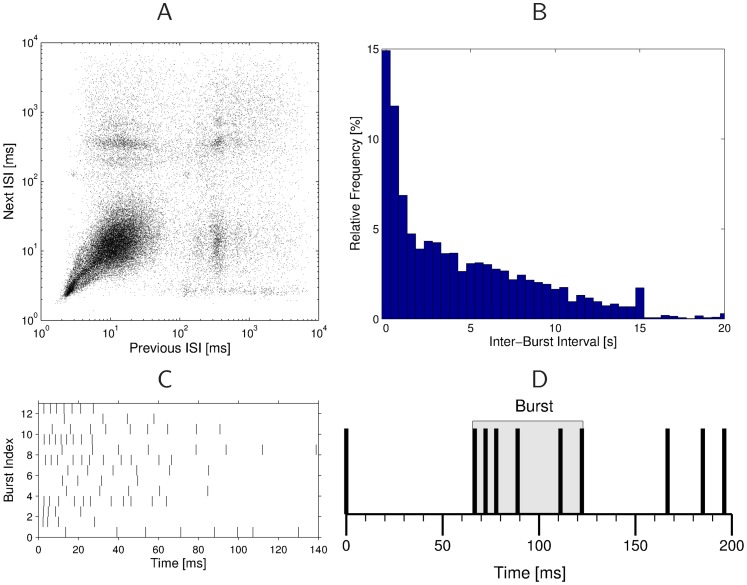
Analysis of bursts extracted from the spike data. **A**) The joint interspike-interval plot for a single preparation indicates the presence of bursts by a large cluster of points in the lower left corner, which represents periods of fast firing, and clusters of points in the upper left and lower right corner, which indicate onsets and offset of bursts. **B**) Histogram of inter-burst intervals (bin size: 0.5 s). **C**) 13 bursts extracted from 1 minute of the recording. The set of bursts contains 2 responses to stimulus 3 (bursts 10 and 13), 2 responses to stimulus 4 (bursts 4 and 8), and 9 responses to different sources of environmental noise. **D**) Detection of bursts in spike trains. The 6 spikes in the shaded area constitute a burst, because they are separated by time window of at least 60 ms from the first spike, the interspike-interval is never larger than 30 ms, the burst duration is longer than 8 ms and there are more than 5 spikes.

Artificial acoustic signals, as well as some natural background stimuli caused bursting activity in the nerve cell, i.e. it was firing at a much increased rate compared to its baseline firing activity. We extracted from the continuous recordings in natural habitats all these short time segments in which the omega neuron was bursting. Our criterion for detecting bursts in a continuous stream of spikes required a silent interval of at least 60 ms before the start of the burst, a constantly high firing rate of at least 33 Hz, a minimum duration of 8 ms, and a minimum of 5 spikes within the burst (see Figure 2D, as well as *Burst Detection* in *Materials and Methods* for more details). In Figure 2 we illustrate the analysis of one recording session. From the joint interspike-interval (ISI) diagram in Figure 2A, which shows the duration of the next ISI as a function of the preceding ISI, one can see the presence of bursts in the recordings. By definition a burst is a period of rapid firing, preceded and followed by a longer period of no or low activity. The accumulation of points in the lower left corner indicates that there are numerous periods of rapid firing, which are typical for firing intervals within bursts. The clusters of points in the upper left and lower right corner show that there are also many short ISIs preceded or followed by longer intervals, which indicate the onsets or offsets of bursts. The intervals between bursts display no clear pattern, but the histogram of inter-burst intervals in Figure 2B shows that most intervals are short, and the frequency of longer inter-burst intervals decays. In Figure 2C we plotted the bursts contained in one minute of recordings in the original order in which they appeared. Looking only at the raw data it is not immediately clear which bursts belong to a common cluster, and although the bursts are from relatively close time points, there is no visible structure of bursts in response to environmental noise.

### Characteristics of Acoustic Discrimination in Natural Habitats

In the recording shown in Figure 1C, each burst of action potential activity before sunset was caused by a stimulus. A detection criterion based on bursts of action potentials or the corresponding increase in spike rate would give “hits” in term of signal detection [25]. Indeed, in all cases when there was an acoustic signal during the experiment at 17.00 hrs, there was bursting activity in the nerve cell and there was no, or only single spike spontaneous activity when a signal was absent, therefore there were no “misses” or “false alarms” respectively.

After sunset, however, this ideal situation for signal detection changed due to the strong increase in background noise. The same preparation at exactly the same position in the rainforest now exhibited high action potential activity (Figure 1D), and only an a priori knowledge of the time of signal presentation (arrow) would allow correct detection of the stimulus. Using the same detection criterion as in the situation before sunset would result in many false alarms (i.e. identifying background noise as signals).

**Figure 3:**
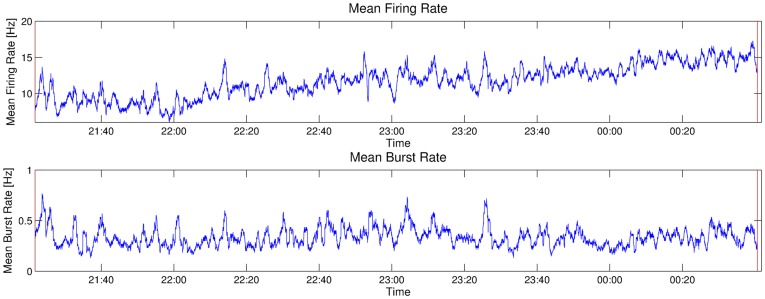
Firing and bursting rates in the natural habitat. Firing (top) and bursting rates (bottom) of the spike activity of the omega-neuron from 21.20 hrs to 0.40 hrs at night in the natural habitat. The fluctuation in both rates is high, but firing and burst rates are correlated with a correlation coefficient of 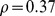. The mean firing rate over the entire night is 11.5 Hz, and the mean bursting rate is 0.33 Hz. (Bin size: 1 sec for firing rate, 100 sec for bursting rate).

Figure 3 shows firing and bursting rates in one recording over a longer time period after sunset, and it is obvious that while the recordings are very stable over multiple hours, there are considerable fluctuations on shorter time scales, mainly due to background noise. In this preparation, firing rates vary from 7 Hz to 17 Hz over the time period of 200 minutes of recording, and burst rates vary from about 0.2 to 0.8 Hz. The curves for firing and bursting rates are visibly correlated (correlation coefficient 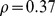). Artificial stimuli are only played every 10 seconds, and preparations typically respond with a single burst to these signals. From the fact that the bursting rate is always greater than 0.1 Hz, one can see that a large majority of the bursts result from background noise (in this recording 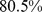 of the bursts are noise bursts).

Apparently, analyzing neural signals recorded under natural conditions poses different challenges in comparison to laboratory experiments, but yields different and more realistic results. The background noise mainly constitutes the communication activity of different individuals and species of insects, frogs and vertebrates. In our recordings we made sure to place the preparation at a place in the rainforest where no conspecific males were singing. The majority of noise therefore comes from heterospecifics with no behavioral relevance. A second category of noise may be sound originating from predators, such as bats.

Another additional difficulty arises because different noise stimuli arrive at the insect from multiple directions in the azimuth and elevation, due to the complex 3-dimensional structure of the rainforest. Since there is a multitude of simultaneously active senders, the animal receives a variety of sound events, and not all of them are coherent in the time and frequency domain. This is very different from typical lab experiments, in which a single stimulus and eventual background noise are broadcast from the same or opposite sides of the animals.

### Stereotyped Response of the Omega Neuron to Acoustic Stimulation

**Figure 4:**
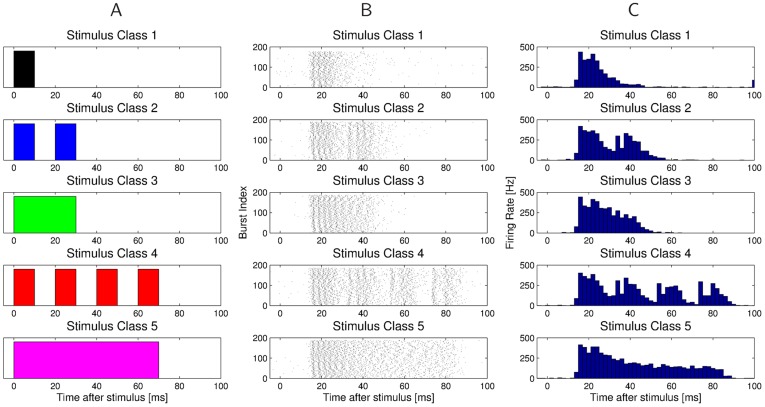
Stimulus aligned responses for a single preparation. **A**) Sketch of the five artificial stimuli. **B**) The structure of the burst spike trains in response to artificial stimuli becomes visible if they are aligned to the stimulus onset. **C**) Peri-stimulus time histograms (bin size: 2 ms) for the five classes of artificial stimuli.

**Figure 5:**
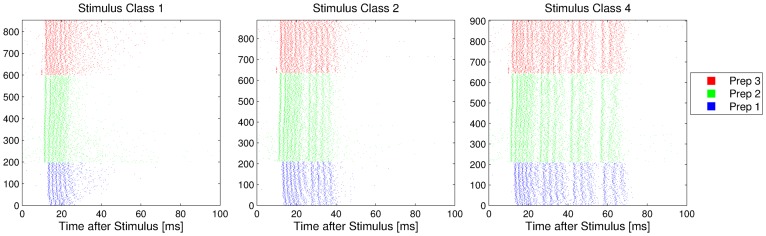
Bursts in response to three artificial stimuli. Recordings are from three different insect preparations (marked by different colors), and bursts are displayed aligned to the stimulus onset. Only classes 1,2, and 4, were played at those recording dates. One can see a clear similarity of the responses, but also different latencies and variabilities of firing.

The structure of bursts in response to the artificial stimulus classes from Figure 1B becomes visible if one aligns the bursts to the onset of the stimulus. For a single preparation, Figure 4 shows the bursts that follow these stimuli as spike train plots and PSTHs, respectively. Considering that the environmental noise before and during the presentation of the stimulus is very strong and inhomogeneous, the responses of the omega neuron to the same stimulus are remarkably similar. The same holds for responses of different preparations from different sessions to identical stimuli, which is illustrated in Figure 5. In those recording sessions from November 2003, only stimuli from class 1,2, and 4 were presented. Also here one can observe that the stimulus aligned firing patterns are qualitatively very similar, with slight variations in the latency of the bursts, or the variability of firing.

### Identification of Burst Patterns with Unsupervised Clustering

After extracting all bursts from the recordings, we used a variant of the spike-time metric by Victor and Purpura [15]–[17] to compute similarities between different bursts (see 6A, B and *Materials and Methods*). This similarity measure then served as the basis for clustering spike trains into homogeneous groups, and assigning a representative cluster *exemplar* to every group, using the affinity propagation algorithm [18]. This procedure is completely unsupervised, based only on the similarity of bursts, and not on labels assigned to the bursts. It creates a variable number of groups of bursts, with the goal of maximizing the similarity of bursts (with respect to the spike-time metric) within each group, and minimizing the similarity between different groups. The algorithms are described in detail in the Materials and Methods section.

For the first experiment we used recordings in which five artificial stimuli were broadcast to the preparations in their natural habitat after sunset. The five artificial stimuli used for playbacks differed in duration and temporal structure (see Figure 1B). Bursts that occurred at the time of the onset of the artificial stimulus were labeled with the class of the associated stimulus. The labels of the bursts were only used for evaluation purposes, but were not provided to the clustering algorithm.

**Figure 6:**
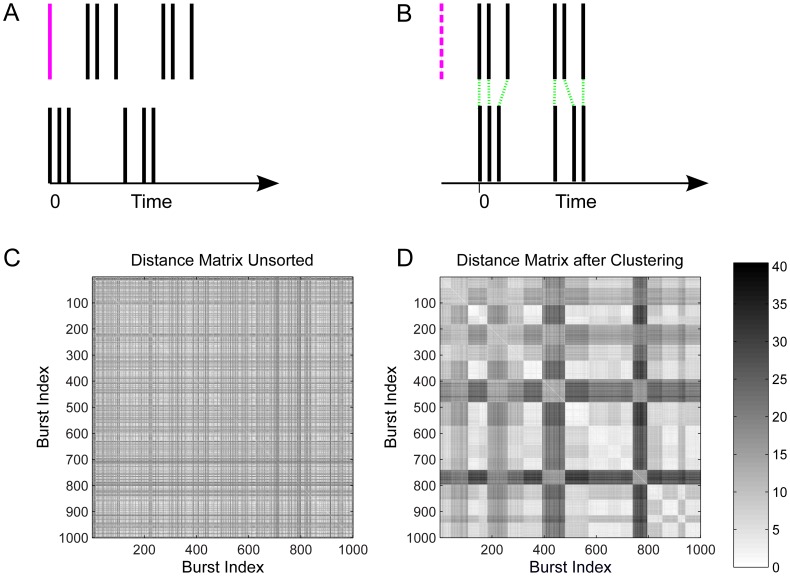
Illustration of modified spike metrics, and spike train distance matrices. **A, B**) Illustration of the burst-shift operator. In **A**, the two bursts appear to be very different under the standard spike-time metric from [15], because there is a single (purple) spike before the pattern of two spike triplets, and so all subsequent spike times are shifted. The burst-shift operator in B deletes the first (purple dashed) spike and re-aligns the new first spikes of the two bursts. The distance between the two spike train then results from a relatively cheap series of spike shifts (green dotted lines), plus the cost for deleting the initial spike. **C, D**) Distance matrices for a single preparation in the natural habitat before (**C**) and after clustering (**D**). Light pixels indicate high similarity of bursts, whereas dark pixels show larger distances. The clustering process leads to the clear formation of groups of similar bursts.

The result of the clustering is illustrated in Figure 6C and D. Here the distance matrix is shown before and after the clustering process (dark indicates large distance). Before the clustering, the distance matrix for 1000 randomly picked bursts is displayed, where the ordering of the burst indices corresponds to the order in which they were recorded. The clustering process rearranged the order of the bursts, by grouping them into homogeneous clusters, which are displayed in the arbitrary order that is produced by the affinity propagation algorithm (see *Materials and Methods*). One can clearly recognize these groups from the distance matrix, by observing blocks that have low inter-cluster distance, and larger distance to other groups of bursts.

### Burst Patterns in Response to Artificial Stimuli and Noise

**Figure 7:**
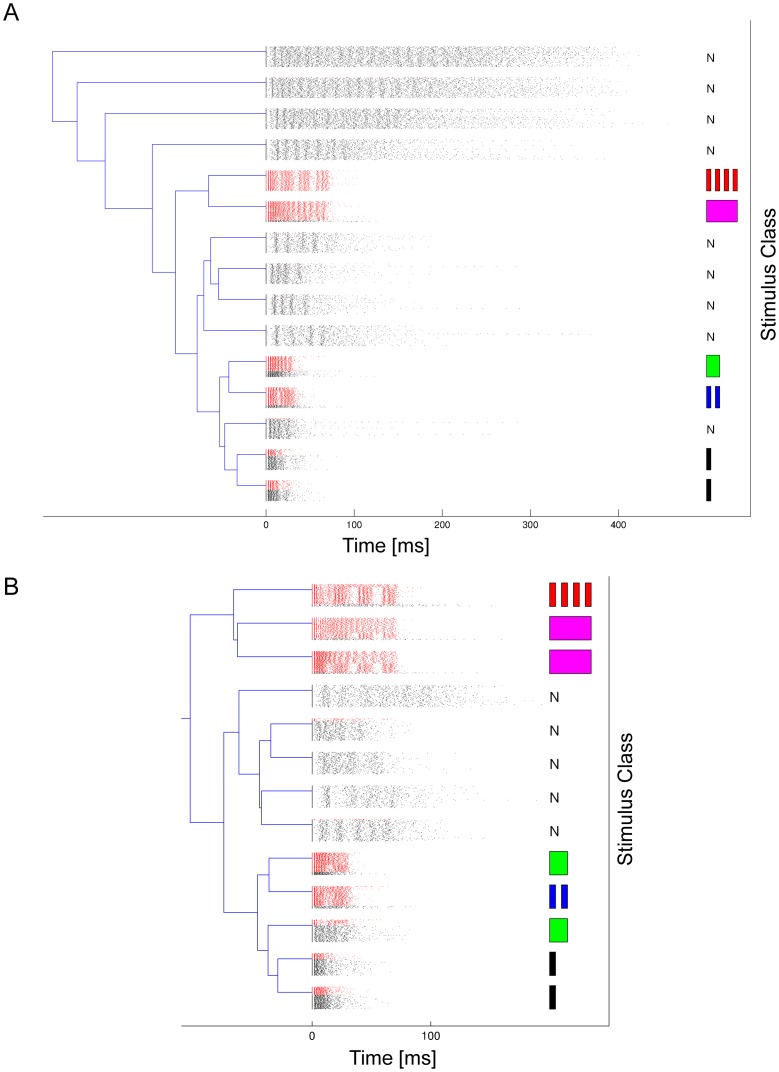
Clusters of bursts from two recordings in the natural habitat. **A**) and **B**) show clusters of bursts obtained from two different preparations on different recording dates. Bursts associated with artificial stimuli are plotted red, bursts associated with noise are plotted in black. On the right is an illustration of the stimulus that is assigned as label to this cluster, or *N* if the cluster mainly consists of bursts is response to noise signals. The clusters are arranged hierarchically, grouping clusters with similar exemplars together. Longer and more structured bursts form more homogeneous groups than bursts after short pulse signals (e.g. clusters at the bottom of diagram B). Clusters in A) contain between 140 and 501 bursts, and between 145 and 328 bursts in B).

Figure 7 shows the groups of bursts resulting from unsupervised clustering, plotting bursts following stimuli in red, and bursts as a result of background noise in black. On the right side we plot a sketch of the stimulus that is assigned as label to this cluster (or *N* if the cluster consists mostly of noise bursts). This label was determined as the stimulus class with the highest percentage of bursts in this cluster relative to its average frequency of occurrence in the whole dataset (see *Materials and Methods*). This avoids a bias towards assigning clusters to noise stimuli, which are four times as frequent as bursts in response to the 5 artificial stimuli.

The labels of some clusters are very homogeneous, in particular those in the upper part of the dendrogram with clusters of long and relatively unstructured bursts, which are almost exclusively bursts in response to background noise. These bursts are grouped together because they have a similar mean firing rate and a similar number of spikes, although their firing patterns do not exactly match. Two other groups of homogeneous clusters are comprised almost exclusively of bursts in response to two artificial stimuli (classes 4 (a four-pulse-stimulus) and 5 (a pulse of 70 ms duration) in Figure 1B); only rarely do we find in the same cluster bursts not elicited by these stimuli. In some cases, however, the unsupervised clustering algorithm produced inhomogeneous clusters, which include both bursts in response to stimuli as well as bursts in response to the background noise. This is true for the two bottom clusters in Figure 7A, which contain most of the bursts in response to the 10 ms pulse (stimulus 1), but also for two clusters with bursts in response to the 30 ms pulse (stimulus 3) and the two-pulse stimulus (stimulus 2), which cluster together with background noise bursts. Figure 7B shows a clustering for another recording with a different preparation and night, and again it can be seen that bursts following one of the longer or more structured artificial signals (classes 4 and 5) fall into more homogeneous clusters than bursts after stimuli with shorter pulses. Bursts following stimulus 1 are again mostly clustered together with noise bursts.

A possible explanation for this is that stimuli 1–3 are similar to signals that can be naturally found in the acoustic background noise of the rainforest, e.g. calling songs produced by other bushcrickets, whereas stimuli 4 and 5 are purely artificial and never occur in the background. One can further observe in both plots that there are some very homogeneous clusters of bursts with precisely timed firing patterns in response to unidentified background noise events. Altogether this illustrates the difficulty of the auditory discrimination problem for the bushcricket under real world conditions. Short and/or unstructured stimuli may lead to false alarms from background noise signals. On the other hand, the discriminability of stimuli can be greatly improved by using more complex temporal structure, such as the patterns in stimulus classes 2 and 4. Our current method finds individual burst patterns that could serve as basic blocks for encoding more complex stimuli, or features of stimuli, in sequences of bursts, which will be an important topic for future research.

Occasionally, bursts for the same classes of stimuli are distributed into different clusters (e.g. class 1 in Fig. 7A and B, class 3 in Fig. 7B, or class 5 in Fig. 7B). Since the clustering algorithm does not know about the labels, it does not attempt to avoid this effect, if it can be explained by the model. Therefore it is possible that the same stimulus leads to a single cluster in one experiment, and two or even more in another. The biological interpretation of this effect might be that different variants of the same stimulus can be encoded by different clusters, e.g. due to different background noise during the presentation of the stimulus. However, we did not record the acoustic background during the experiments, since this is technically very difficult to achieve in a real-world environment with complex 3-dimensional structure like the rainforest, and a direct mapping between sound recordings and the acoustic stimulus sensed by the animal is in general not possible.

### Discriminability of Artificial Stimulus Classes

Figure 7 demonstrates that bursts in response to particular classes of artificial stimuli form very homogeneous clusters, e.g. bursts in response to the four pulse pattern (class 4). On the other hand, some stimulus evoked bursts are mostly mixed together with bursts in response to background noise in the habitat, or bursts in response to different stimuli. We evaluated this separability property of stimulus evoked bursts over data from six recordings sessions, of which three used all 5 stimulus classes, and three contained only stimuli of classes 1,2, and 4.

As a measure of homogeneity we used the average conditional entropy of class labels for every cluster (see *Materials and Methods*). The conditional entropy in our case is low if knowing the cluster label reduces the uncertainty about the classes of bursts that are found within the cluster. So in the ideal case there should be only one class of bursts in every cluster (which means zero entropy).

**Figure 8:**
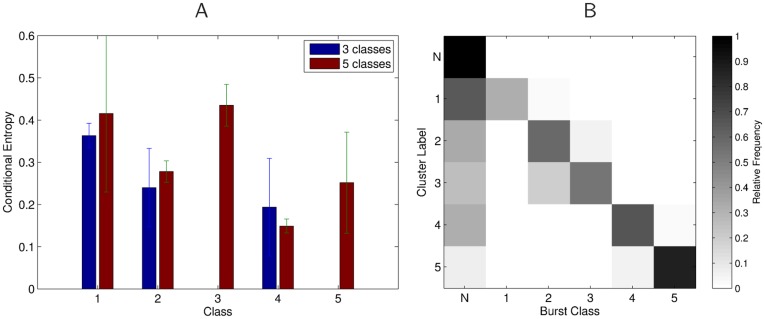
Analysis of separability of bursts in response to artificial stimuli from other stimulus classes. **A**) Conditional entropy of class labels given the cluster indices, averaged over six recording sessions, three in which all 5 stimulus classes were used, and three in which only classes 1,2, and 4 were used (errorbars denote standard deviations). Classes of bursts with lower conditional entropy form more homogeneous clusters. Artificial stimuli that consist of temporally more structured and/or longer stimuli (classes 2,4, and 5) are better separable from noise or other stimuli than single pulse stimuli (classes 1 and 3). **B**) Confusion matrix for assigned cluster labels vs. actual labels of bursts. In every row we plot the average relative frequencies of burst labels occurrences in clusters that were assigned to one of the classes *N* ( = Noise) or 1–5. One can see that most mistakes are due to noise bursts assigned to one of the artificial stimulus classes. Also bursts in response to classes 2 and 3 are sometimes clustered together.

Figure 8A shows the average conditional entropy individually for every class, where the average is over all recordings sessions in which those stimuli were used (three sessions for all 5 stimuli, three sessions only for stimulus classes 1,2, and 4). Although these statistics are based on only six recording sessions, and the standard deviations are large, one can observe the same trend that was qualitatively visible from Figure 7. The average conditional entropy is low for classes of bursts in response to long and/or temporally structured stimuli (classes 2,4, and 5), and higher for the single short pulses of classes 1 and 3. This indicates that classes 2,4, and 5 can be better discriminated from other artificial or background signals than the single pulse stimuli. Due to the limited amount of available data, these results are statistically not significant, and more measurements would be required.

In Figure 8B we show the confusion matrix that results from assigning class labels to each cluster (see Materials and Methods). One can see that the major source of errors are noise bursts being assigned to clusters that represent artificial stimulus classes. Classes 2 and 3 are also sometimes clustered together, whereas classes 4 and 5 are mostly found in homogeneous clusters.

### Similarity of Burst Patterns in Different Preparations

The two clustering results in Figures 7A and 7B indicate that similar clusters of bursts can be found in both recordings, even though the recordings stem from different preparations and different recording sessions. This is even more remarkable if one considers that the background noise in the natural habitat is far from constant over the recording periods, since different sound sources may be present and located at different positions in comparison to other recording sessions in different nights, and even years. To further analyze this similarity of neural responses, we searched for burst clusters from one recording session that have corresponding clusters of bursts in different recording sessions with similar firing patterns. Starting from the previously computed clusterings of bursts from single recording sessions, the spike-time metric described in Methods was used to calculate all distances between the cluster exemplars from different sessions. For every cluster in one recording the cluster with the closest matching exemplar in the other recording was then selected. Since we have observed that firing patterns are characteristic for the acoustic stimulus that they encode, a high similarity of two clusters in different sessions would suggest that the contained bursts are responses to the same or a similar type of sound source. For comparison, and for understanding whether the matching is based on the firing pattern or purely on statistical properties like average firing rate and duration, we also computed the average spike-time metric between bursts from the two clusters if every spike train was replaced by an inhomogeneous Poisson spike train, whose time-dependent firing rate profile was given by the average population rate of all bursts in the cluster. In all experiments we observed that the distances between the Poisson spike trains were always substantially higher than the matching distances of the cluster exemplars. This indicates that the firing patterns of bursts inside a cluster are much more precise than the Poisson spike trains.

We first analyze the similarity of clusters in response to the same stimuli under two different acoustic conditions. For several preparations we recorded the response to artificial stimuli in the laboratory, and for others outdoors. Obviously these recording conditions are very different, because the majority of bursts (around 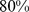) in outdoor recordings stem from background noise, while in the laboratory bursts occur almost exclusively in response to artificial stimuli (only 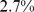 of all bursts result from spontaneous activity).

**Figure 9:**
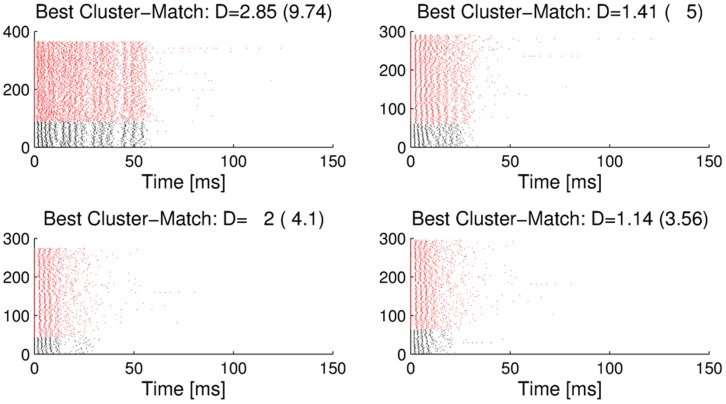
Matching of laboratory burst clusters to outdoor recordings. Clusters of bursts from laboratory recordings (black), matched to clusters of bursts from outdoor recordings (red). Four examples of matched clusters (in response to stimulus classes 4 (top left), 3 (top right), and 1 (bottom left and right) are shown. For these laboratory clusters, closely matching clusters are found in the outdoor recordings. *D* defines the distance between the exemplars of the two matched clusters under the spike-time metric. As a comparison, the numbers in parentheses give the average distances between Poisson spike trains with identical time-varying firing rate profiles.

In Figure 9 the clusters of bursts found in a laboratory experiment, in which only artificial stimuli of classes 1,3, and 4 (see Figure 1B) were broadcast, were matched to clusters from outdoor recordings (the burst labels are not used for the matching). As can be seen from the comparison of clusters, there are very close matches of laboratory-burst clusters to clusters from outdoor recordings. On the other hand, the responses to the four-pulsed stimulus in the laboratory condition reveal a more precise timing of spikes within the bursts compared to the responses recorded outdoors. This indicates that the specific acoustic conditions of the noisy nocturnal rainforest caused some changes in this timing within bursts. Still, even in the presence of this strong distracting noise, the omega neuron responds with a very similar pattern, that significantly simplifies the decoding tasks for higher processing areas.

**Figure 10:**
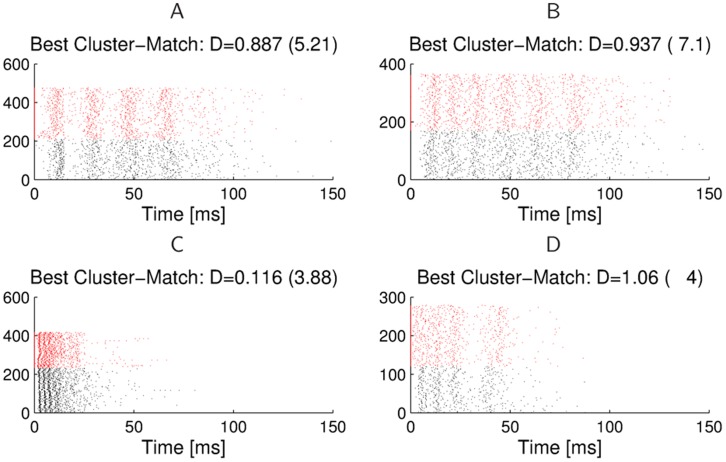
Clusters of bursts from two different outdoor recordings and their best matching cluster. The examples show matches for burst clusters in response to natural background noise. *D* defines the distance between the exemplars of the two matched clusters under the spike-time metric. The numbers in parentheses give the average distances between Poisson spike trains with identical time-varying firing rate profiles.

In a similar way we matched clusters of bursts from different outdoor recording sessions, in which the activity of omega neuron from different animals was recorded at different nights (sometimes in different years). The examples of cluster matching results in Figure 10 show that also under these conditions it is possible to find close matches for some clusters of bursts. Comparing the similarity indices *D* in Figures 9 and 10 would indicate that some of the cluster matches between different animals in different outdoor recording conditions are closer than the cluster matches between outdoor and laboratory recording conditions. The reason for this might be the lower number of spikes under lab conditions, due to the complete absence of noise. The matching distance is substantially lower than the distance of Poisson spike trains with identical statistics, which indicates that the precision of firing in both preparations is higher than can be explained by a stochastic, purely firing-rate based model.

On the other hand, the cluster matching procedure also revealed several clusters of bursts for which no good match in the other recording session was found. This holds in particular for clusters of long bursts without clear temporal structure, which typically result from background noise. Such clusters have a substantially higher distance *D* to the best matching cluster, which is due to the fact that these bursts include more spikes, and so more shifts or insertions may have to be made in order to transform one spike train into another. Bursts within these clusters do not show the precise temporal signature that could be observed in the previous analysis, and could arise e.g. in response to senders in the background with temporally extended calls and low amplitude modulation. Such long stimuli also have a higher probability of being interrupted by another stimulus of higher behavioral relevance. It is therefore not unexpected to find higher variability in these burst patterns, both within the same preparation and between different preparations.

### Similarity of Burst Patterns in Simultaneous Recordings of Homologous Cells

**Figure 11:**
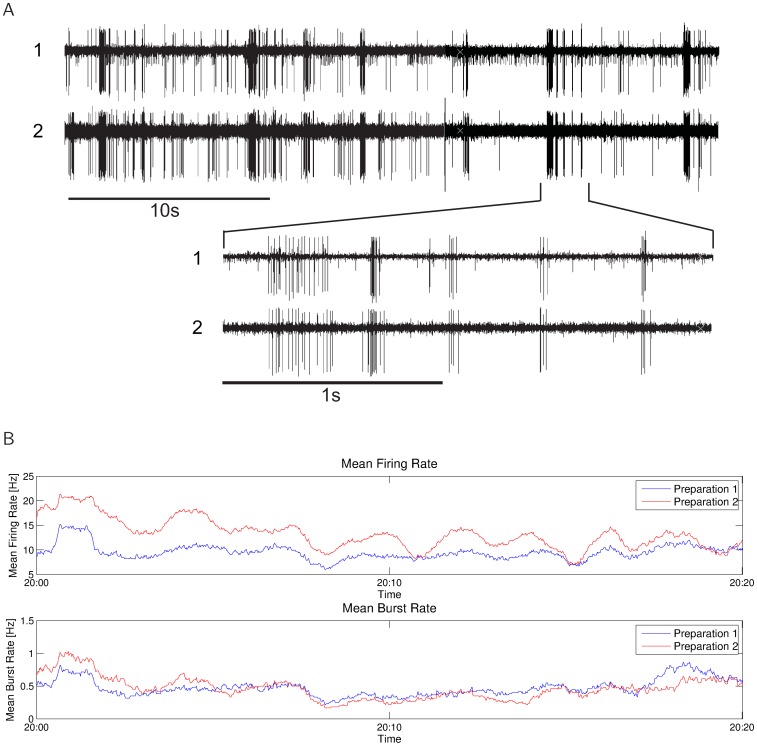
Firing and bursting during simultaneous recordings. **A**) Short sequence of the original spike recording of both cells recorded simultaneously. **B**): Firing and burst rates of both cells for a duration of 20 minutes. The firing rates of preparation 1 and 2 are correlated with a correlation coefficient of 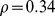. The burst rates are correlated with 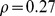. Mean firing rates over the entire 20 minute recordings are 9.98 Hz (preparation 1) and 12.20 Hz (preparation 2), and mean bursting rates are 0.53 Hz and 0.41 Hz respectively. (Bin size: 1 sec for firing rate, 100 sec for bursting rate).

**Figure 12:**
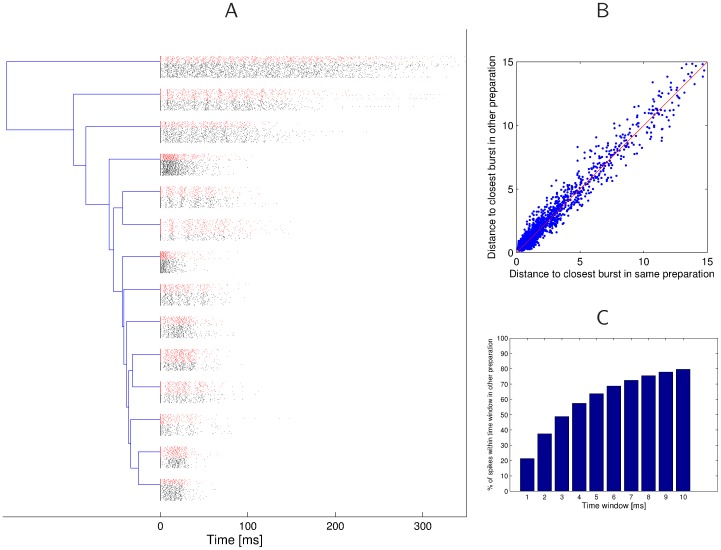
Similarity of burst patterns in two simultaneously recorded preparations. **A**) Clusters for the aggregated bursts of two omega-cell preparations recorded simultaneously in their natural habitat; no broadcast of artificial stimuli. Bursts from preparation 1 are drawn in red, and bursts from preparation 2 in black. Every cluster contains about half of its bursts from one preparation. **B**) Spike-time distance of the most similar burst in the same preparation in comparison to the most similar burst in the other preparation. The clustering of points around the diagonal shows that for every burst in one preparation the distance of the closest match in the same and in the other preparation are almost identical. **C**) Similarity of spiking times within bursts. For every spike at time *t* in a burst in one preparation we compute for different time windows 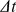 how often there is a spike within a burst in the other preparation in the time window 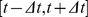. We find that 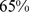 of all spikes have a corresponding spike in the other preparation within a 5 ms time window, and 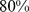 within a 10 ms time window.

In the previous section we compared the similarity of burst activity in the omega-neuron between lab and outdoor recordings, or between different cells in different nights. The “biological microphone approach” offers in addition one unique opportunity to test the power of our method, by comparing the burst responses of omega cells from two different preparations recorded simultaneously, and placed next to each other, so that they experience the same acoustic events. For the experiment presented in Figures 11 and 12 the two preparations were placed in the nocturnal rainforest, at a distance of about 10 cm from each other, so that they were exposed to the same acoustic environment. Prior to these recordings, the threshold of each omega-cell in response to a pure tone, 20 kHz stimulus was determined in the laboratory, and one preparation was 5 dB less sensitive compared to the other preparation. In this experiment, no artificial sound stimuli were broadcast to the preparations, so all bursts had been elicited as a result of background noise alone. Figure 11A shows a short sequence of the original spike recording of both cells, and in Figure 11B the firing and burst rates of both cells are illustrated for a sequence of continuous 20 minutes of recording. The gross firing and bursting pattern of both cells is rather similar (Figure 11A), although the less sensitive cell exhibits a reduced firing rate (Figure 11B). The firing rates are actually correlated with a correlation coefficient of 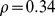, the bursting rates are correlated with 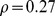.

Even though the firing behavior of the two omega-cells is slightly different due to the threshold difference of 5 dB, one should find similar spiking patterns in the bursts, as the two preparations had been exposed to the same background noise. For both preparations the bursts were extracted, which results in 936 bursts for preparation 1 and 726 for preparation 2. We used the affinity propagation algorithm to find clusters in the aggregated set of bursts from both preparations. Figure 12A shows the resulting cluster dendrogram, where bursts from the first preparation are drawn in red, and bursts from the second preparation in black. Every cluster contains bursts from both preparations, and the relative frequencies of bursts originating from either preparation are balanced. On average, 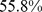 of the bursts in every cluster are from preparation 1, which is a result of the higher number of bursts extracted from preparation 1. The minimum percentage of bursts from preparation 1 in any cluster is 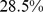, and the maximum percentage is 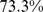. We also show in Figure 12B for every burst in the two preparations the spike-time distance of the closest matching burst in the same, and in the other preparation. One can see that those distances scatter around the diagonal, which indicates that for every firing pattern in one preparation we can find a similar one in the other, which is not a worse match than other bursts in the same preparation. If one looks at the exact spike times of the two preparations, one can see in Figure 12C the relative frequency that within a time window of 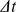 before and after a spike in a burst in one preparation there is a spike in a burst in the other preparation. In more than 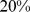 of the cases there is a spike within 1 ms, and in 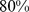 of all cases there is a spike within 10 ms. This shows that even though the two preparations do not fire at exactly the same time, they will frequently fire within a short time window after each other.

All these results suggest that there are no firing patterns that are uniquely found only in one preparation, but not in the other. Even though individual burst responses of the two preparations at any time may show stronger variations, the global bursting patterns in response to the same acoustic background are very similar for different preparations. This result provides further evidence for the remarkably well preserved burst-coding mechanism in response to complex real-world auditory stimuli that is shared by individuals of this species. In the following Discussion we will analyze the significance of these results for communication under real-world conditions.

## Discussion

### Coding Problems for Stimuli in the Natural Environment

For the two major tasks of sensory systems of object classification and discrimination the central nervous system needs to interpret the ongoing afferent spike activity. Consistent with a number of previous studies on sensory coding in different modalities we view short bursts of action potentials as the basic units for the representation of information. The importance of bursts, in contrast to single spikes, has been discussed in the context of the efficiency of synaptic transmission and thereby synaptic plasticity [7], in the selective distribution of information to different target neurons [26], or the dynamics of encoding behaviorally relevant stimulus features [4], [27]–[30]. Bursts can be viewed as robust symbols for the neural coding alphabet; they can carry information in their duration, the number of spikes, or the exact timing of the firing pattern, and specifically tuned synapses may read out such a code easily [31].

However, classification and discrimination are severely impaired by variation in afferent spike trains, either as a result of intrinsic noise in nervous systems, or external noise resulting from interactions of the stimulus with the transmission channel. A further source of variability of high relevance for a receiver is introduced as a result of small differences in the features of signals from different sources, such as the signals of mates. Ronacher et al. [9] reviewed the sources of spike train variability and the associated problems and constraints for producing adaptive behavior in grasshoppers. In the case of the auditory system, a further problem results from the background noise of many natural environments, so that relevant stimuli (and stimulus variants) have to be discriminated from irrelevant background noise. Research in the past decade demonstrated that the auditory system of many animals evolved mechanisms to cope with such noise [32].

In all comparable studies of neural coding with bursts in the past, preparations were studied under controlled conditions in the sound proof lab. The studies by [33] in grasshoppers and [34], [35] in songbirds, for example, played back previously recorded songs of conspecifics, and investigated how the individual songs can be discriminated from the neural response of auditory receptor cells [33] or cortical neurons [34], [35]. Another common approach to study neural coding is to use time-varying (often random) artificial stimuli, and measure how accurately the whole stimulus, or certain features of the stimulus, can be reconstructed from the neural response in-vitro or in-vivo [27]–[30], [36], [37]. The main advantage of these methods is that the experimenter has full control over the stimuli (e.g. to modify their duration or amplitude), and eliminates distractor signals. On the other hand, the complete absence of environmental noise creates an artificial scenario for the receiver, which may hide the influence of potentially important selective attention mechanisms [38], [39].

In contrast to these previous studies we investigate here the most realistic possible scenario for sensory coding, using stimuli and neurophysiological recordings directly in the natural habitat of the organism. We did not attempt to correlate the bursts in the omega neuron with simultaneously recorded sound stimuli, since it is almost impossible to characterize the acoustic environment and all its relevant features with technical sensors like microphones, for several reasons. First of all, high-frequency microphones would be needed to record in the frequency range relevant for the bushcricket, with absolute sensitivities about 20 dB less than the insect ear. Then, an array of microphones would be required to characterize the direction of a sound, but each single microphone has a much stronger directionality, compared to the insect preparation. Even if one provided all this technical effort, it would not yield the desired results, simply because the insect hears something different [13], [14]. A burst in the omega neuron would therefore often find no counterpart in the sound recording. We are fully aware of the fact that the selected neuron is a first-order local interneuron that does not provide information to higher brain centers via an ascending axon. We do not argue, though, that the omega-cell in katydids represents the neuronal element for signal discrimination. Rather, we used this cell because it integrates most of the activity of auditory receptors in the ear, as an indicator for the information that can be extracted from the timing of spikes in its discharges. It has been shown, however, that in the auditory pathway of katydids such interneurons do exist, with broadband tuning and tonic responses similar to the omega-cell [40].

In addition to the new methodological approach for recordings in natural habitats, this paper explores the use of a new method for unsupervised clustering of data, based on probabilistic inference. We can thus ask how much information an organism can extract from bursts without ever being told by a postulated “supervisor” which type and which variants of bursts are characteristic for a particular stimulus.

It is now widely agreed that the encoding of stimuli by sensory neurons is adapted to the statistics of stimuli in the environment in which an organism lives [41]. Variants of Barlow’s efficient coding hypothesis, for example, have been studied for over 50 years [42], [43]. The hypothesis suggests that stimuli that occur frequently in the natural environment are encoded particularly efficiently by sensory neurons. Under this hypothesis the benefit of particular coding schemes for natural stimuli can be quantified with tools from information theory. Early studies for the visual system [42] have also suggested that early sensory neurons reduce redundancies in the input, in order to use available computing resources most efficiently. A similar argument was made in the “matched filter hypothesis” in that rather peripheral ‘matched filters’ may relax the nervous system from computational strain [44], [45]. A more recent review of the implications of the efficient coding hypothesis for visual systems can be found in [46]. As is pointed out in this review, efficient coding of natural stimuli should not be studied in isolation, but must also take into account the robustness of neural representations to noise in the environment and stochastic processes at the neuronal level.

The efficient coding hypothesis has recently been challenged by [47], using a comparative study of homologous neurons in two grasshopper species. They demonstrated that stimuli of high relevance for one species were processed in the afferent auditory system of the other species in exactly the same, quantitatively indistinguishable way, although being “meaningless” in terms of any behavioral relevance (for a similar finding see [48]). This suggests that neuronal elements of the sensory system have been strongly conserved during the evolutionary convergence of the two species. Similarly, in our study we used as a model system a single interneuron, the so-called omega neuron, which has been identified in all species of crickets and bushcrickets so far studied [19]. We do not argue, therefore, that the burst coding we found in our study demonstrates specific adaptive properties of the species under study. Rather, we chose to use this insect preparation because of the simple architecture of insect auditory pathways, and their remarkable precision and discrimination abilities in general [49]–[51]. A further reason was that the interneuron is part of an early processing stage, directly postsynaptic to almost all 20–40 auditory receptor cells in the ear [23], so that it integrates signals from almost all receptor cells and a wide range of carrier frequencies from less than 10 kHz far into the ultrasonic range. Thus, monitoring the activity of the cell directly in the animals’ own environment provides information about the complete acoustic input of the animal under study, encoded in its spike activity. Therefore, our study is among the first to investigate sensory coding under the natural environmental conditions of an organism, instead of idealized lab conditions.

### Detecting Spike Patterns with Unsupervised Learning

We have used clustering as an unsupervised tool to detect burst patterns in raw neural recordings. Bursts are characterized by their similarity to all other bursts, measured by a spike metric [15]–[17]. In a good clustering, bursts that are grouped into the same cluster are similar to each other, but dissimilar to bursts in other clusters. The result of a clustering therefore provides a characterization of different spike patterns that occur frequently in the recorded spike train. Our clustering is based on exact firing patterns, but it is also possible to compute clusterings based on numerical features extracted from bursts (such as firing rate, duration, …), or by binning spike trains into discrete time windows [37]. The disadvantage of these methods is that substantial information about the temporal structure of the bursts is already lost by replacing the exact pattern with a lower-dimensional feature vector. In our non-parametric approach we work with the exact spike times, and only lose information by going from the original dataset to the matrix of spike-train distances, which usually preserves most of the structure in the data.

The results obtained with unsupervised clustering demonstrate t’hat information in the omega neuron is not simply encoded by the presence or absence of a burst. The reliability of burst coding in the time domain was very high, so that bursts in response to one of the presented stimuli clustered differently from bursts in response to background noise. However, this was only true for longer bursts, or bursts resulting from repetitive, temporally structured stimuli (e.g. class 4; Figure 1B). It was difficult to distinguish neural responses to short pulse signals from acoustic background noise, since they often clustered together with bursts induced by the background. This would indicate that reliable coding of short signals with little amplitude modulation is severely impaired under high background noise conditions of the nocturnal rainforest. For the communication of the investigated species, and a number of other katydid species (in particular the Phaneropterinae) this has important implications. In our analysis of male calls of more than 20 species the majority uses sound pulses of less than 10–20 ms in duration, so that burst responses to these pulses would likely cluster with bursts in response to heterospecific signals, as shown for those of *D. gigliotosi*. Moreover, the coding task is substantially more difficult in species with extreme low signal duty cycles. In the case of *D. gigliotosi*, one short sound pulse is produced once every 10 seconds, and in the most extreme case of another phaneropterine katydid this is one for every 3 minutes. Our data indicate that in the long periods between two signals there are many sensory bursts elicited by the background of high similarity with the bursts induced by the signals. In most species, however, these pulses are either produced in a species-specific repetitive way or differ in some finer details of amplitude modulation.

Evidence for the importance of bursts for insect auditory codes comes from in vivo studies in grasshoppers. Studies in *Locusta migratoria* receptor neurons under lab conditions revealed that a neuronal code based on the burst onset time and type (defined by the number of spikes in the burst) preserved most of the information about acoustic stimuli [52], [53]. These studies also showed that the stimuli encoded by different burst types are significantly different from what would be obtained by a combination of single-spike triggered averages for the spikes within a burst. Creutzig et al. [54] showed that burst responses of the AN12 acoustic interneuron of *L. migratoria* and *Chorthippus biguttulus* are preferentially triggered by the onset of syllables of communication signals. Furthermore, the number of spikes inside a burst can serve as a timescale-invariant encoding of behaviorally relevant information, such as the duration of the pause preceding the syllable.

Depending on the individual preparation, the particular recording session, and the parameter settings, the clustering revealed a variable number of different clusters of bursts over the period of some hours (see e.g. Figure 7). If we assume that these different bursts are representations of different signalers, the data give some hints for the requirements of the discrimination task(s) of insect receivers. The number of variants to be discriminated (and thus the difficulty of the task) will affect the speed and accuracy with which it is solved, and can result in speed-accuracy trade-offs in animal decision making [55]. First, they need to distinguish conspecific mates and rivals from heterospecific (irrelevant) signalers. This task is probably the easiest, because the amplitude-modulation of most insect calling songs differ from species to species considerably [56], as should be the case with their representation at early stages of the afferent sensory system. Nevertheless, the transmission channel for sound can impose strong fluctuations on these amplitude modulations [13], [57], [58], an effect which increases with distance, so that even this classification task is not without any problems. Furthermore, the probability of signal interference increases with the number of signalers obscuring important features necessary for species recognition. We have seen in our comparison of bursts always recorded at the same position in the rainforest, but at different nights (or years), that some bursts cluster very close together (see quantitative values of D). This would indicate that the specific amplitude-modulated signal of the same individual (or species) elicited rather similar bursting activity in the different receivers, so that these species-specific signals appear to be well represented in the sensory system.

The second task, namely the discrimination between different variants of signals produced by different signalers of conspecifics, is certainly more demanding. In their study on the representation of such variants in grasshopper receptors Machens et al. [33] have shown that the precise timing of spikes would indeed allow such discrimination under ideal laboratory conditions. However, in the real world situations the precise timing of spikes will be modified by background noise or transmission effects, so that such signals (and their variants) need not only be classified as relevant and different from the acoustic background, but there is also the need to discriminate strongly against any burst activity as a result of predator action/vocalisation. Acoustic insects face a variety of such predators, and one of the best studied are the defense and avoidance behaviors in response to insectivorous bats [59], [60]. Behavioral studies on crickets indicate that the discrimination of “good” and “bad” (conspecific from predatory bats) is based on categorical perception [61], and is rather reliable, since it is based on input within a low-frequency and an ultrasonic frequency channel. In bushcrickets, such a discrimination based on frequency is impossible, since conspecifics and bats use similar carrier frequencies. Thus, the important information about predators must be based in the amplitude modulation as well, and should be present in afferent bursting activity recorded at night. For example, in the predator detection system of noctuid moths, Waters [62] demonstrated that even intrinsic noise in the form of spontaneous action potentials may reduce the ability of moths to discriminate bat from non-bat signals. He proposed that a moth would only be able to recognize an approaching bat from the repetitious nature of the search calls of a bat. This stimulus feature of echolocation signals was found to be preserved in the spiking response of auditory neurons recorded in katydids that were exposed to natural rainforest noise, a fact that allowed the development of a “neuronal bat detector algorithm” [63].

This points to a need for further refinement of our approach, since the algorithm so far developed does not allow for clustering repetitive bursting activity, which should be elicited by the 7–20 Hz repetition rate of echolocation calls in the search phase of bats. Similarly, many acoustic insects use characteristic repetition of the same basic call elements, which could also not be detected by the presented algorithm. The classes of bursts identified by our method could, however, be used as a starting point for identifying longer burst sequences in hierarchical approaches. We expect that the identified firing patterns of bursts will serve as robust symbols in the neural coding alphabet, and their temporal alignment provides relevant information about the identity, location, and other important characteristics of an acoustic sender.

Most remarkably, preparations from different nights show a very high degree of similarity (as quantified by the similarity index *D*), both for outdoor conditions and recordings in a sound proof room. This indicated a high sensitivity of the algorithm for the details of the temporal firing pattern within bursts, since the same homologous cell in different preparations placed at the same position in different nights may experience the same/similar broadcast signals of other animals, and these elicit a rather similar firing pattern. Precise firing times may thus rather encode important features of the stimulus, especially for stimulus features with high behavioral importance, instead of being artifacts of noise in spike generation mechanisms. In the fly, experiments in visual neurons under lab conditions have shown that the amount of information about the current stimulus carried by temporal patterns is 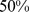 higher than the amount of information carried by firing rate alone [37].

We also found a higher degree of similarity between burst responses to the same stimulus in two different outdoor recordings, compared to responses in the sound proof lab. Whether this can be attributed to the gain-control mechanism of the omega neuron [38], [39], which may be activated by high levels of background noise, and thus alter the finer details of temporal firing within a burst, or whether it simply is an effect of sparse coding in the absence of noise requires further investigation.

### Comparison of Unsupervised Learning Techniques for Spike Data

Spike-metrics, which we use in our approach for computing similarities of spike trains, have been used in a number of other studies for auditory discrimination. [35] and [34] analyzed the time scales at which different conspecific songs could be discriminated by auditory cortical neurons of songbirds under idealized lab conditions. Using the Victor-Purpura [16] and van Rossum [64] spike metrics with different temporal resolutions, they could show that the best performance was reached for spike timing metrics with short time-scales, which emphasize precise firing times in contrast to firing rates. In contrast to our unsupervised approach, their learning framework was based on supervised classification, i.e. template patterns for every sender were known. In a similar approach, Machens et al. [33] used the van Rossum metric [64] for supervised spike train discrimination, and showed that individual calling songs of grasshoppers can be discriminated reliably at the single receptor level, if a metric with high temporal resolution (5 ms) is used.

Finding firing patterns in spike train recordings requires a clustering algorithm which is suitable for this kind of data. Spike trains are not objects in Euclidean space, which is required for basic clustering methods like *k*-means. We have presented one of the first applications of the affinity propagation algorithm [18] to neural recordings. Previous application of affinity propagation have used it for spike sorting [65], for identifying voxels in fMRI data with similar spectro-temporal response properties [66], and for identifying groups of neurons that are either anatomically strongly connected [67], or fire synchronously [68]. However, to the best of our knowledge this is the first time that affinity propagation has been used to detect bursting patterns in spike recordings. Affinity propagation is fast, reliable, and does not require a lot of parameter tuning for finding suitable firing patterns.

Unsupervised methods for finding firing patterns were first used in [69] and [70], using spike metrics and a fuzzy *k*-means algorithm on the Euclidean space of distance vectors between all spike trains. With this method they could identify various spike patterns in response to artificial stimuli for recordings from rats, monkeys, and cats. Their approach of encoding one spike train by the distance vector to all other spike trains is only practical for relatively small datasets, because the feature vectors become larger with every new training example, and clustering becomes increasingly difficult in higher dimensional feature spaces. The last point is not a problem for affinity propagation, because it does not embed the data in a feature space, and instead uses only distances between data points. Memory limitations are still an issue, but in this study we could apply affinity propagation to very large datasets with more than 10,000 bursts, and in [18] there are suggestions for efficient approximations to handle even larger problems by using sparse distance matrices (these approximations were not used in our study).

An alternative information-theoretic method is used in [36],[37], where spike times are binned into 2 ms time windows, and a distribution over binary “codewords” for spike trains preceding common stimuli is computed. The similarity measure is then the Jensen-Shannon divergence between codeword distributions. This method is suitable for experiments under lab conditions, where the stimuli can be precisely timed, and a distribution of responses can be calculated through multiple presentations of identical stimuli. In [36] it was used for studying the neural code of H1 neurons in the fly visual system. Their approach is quite different from our approach towards identifying spiking patterns, because clusters are formed in stimulus space, rather than in response space. It reveals groups of stimuli that cause similar neural responses, rather than firing patterns that occur frequently in the response spike train.

### Conclusion

This study illustrates the importance of studying neural coding under the most natural possible conditions. Many species have evolved mechanisms to perform reliable discrimination and classification of behaviorally relevant stimuli in extremely noisy environments. An understanding of these mechanisms is not possible from recordings in isolated laboratory conditions, and by using completely artificial stimuli and no or artificial background noise. The methods presented in this paper provide a powerful toolbox for analyzing temporally precise neural codes, and understanding robust mechanisms for recognition of sensory inputs under noisy real-world conditions.

We have shown here that different bushcricket individuals in their natural habitat exhibit remarkably similar and precisely timed coding properties in their omega neurons in response to both artificial and natural stimuli in the background. This points to a common intrinsic mechanism in the omega neuron that may facilitate the processing task of higher areas in the insect brain. Using patterns of bursts as primitive elements in higher-level auditory recognition might be a candidate mechanism for solving difficult classification tasks, such as inferring whether a sound in the same frequency band was caused by a conspecific, heterospecific, or a predator, while maintaining the temporal precision to identify differences between variants of conspecific senders.

## Materials and Methods

### Animals and Physiological Preparation

Recordings were obtained from the omega neuron of 27 adult male and female bushcrickets (*Docidocercus gigliotosi*). The methods of the preparation and for obtaining extracellular action-potential recordings of the neuron have been described in detail in [71]. In short, the prothoracic ganglion was surgically exposed in a preparation ventral side up and the tip of an electrolytically sharpened tungsten electrode (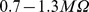 resistance) was inserted into the anterior part of the ganglion, slightly lateral to where the neurite of the omega-neuron crosses the ganglionic midline (see Figure 1A). Then the opening in the cuticle was sealed with petroleum jelly to prevent dessication.

Field recordings were performed on Barro Colorado Island (BCI), located in central Panama within Gatún Lake, part of the Panama Canal, at times after sunset (typically 6 p.m. local time). Data collection took place in February/March, June/July, and November 2002,2003 and 2004. First, the preparation was tested for intrinsic spontaneous activity and for its response to the stimuli without background noise in an anechoic chamber in the laboratory. Background noise level in this chamber was below 30 dB SPL and thus below the threshold of the omega-cell preparation. Five different stimuli were broadcast through a speaker (TW8 spezial) at an intensity of either 10 or 20 dB above the threshold of the preparation. The carrier frequency of the stimuli was constant at 20 kHz, but the number and duration of pulses was different for every stimulus class (see Figure 1B). The rise- and fall-time of all sound pulses was 1 ms. Stimulus intervals were 10 seconds, which is within the range of the naturally occurring intervals in the calling song of this insect [21]. Action potential responses of the omega cell were digitally recorded at a rate of 20 kHz together with the trigger for a stimulus, on separate channels of a data acquisition system (PowerLab, ADInstruments Inc.).

After completion of the control experiments indoors, the preparation with the single cell recording was mechanically stable enough to be transferred to a position within the rainforest about 200 meters from the lab, and fixed at a distance of 2 m from a speaker at a height of 1 m from the ground. The same stimulation regime as in the laboratory was used for outdoor recordings. Since some recordings lasted for several hours (max of 9.5 hrs), at the end of a stimulation regime we controlled for a change in sensitivity of the preparation. If the sensitivity was decreased by 5 dB or more (which happened in four cases), the recording was discarded.

### Burst Detection

The goal of the burst detection mechanism is to extract from several hours of spike recordings those short segments in which the omega neuron is bursting. A burst in a spike train can be qualitatively defined as a short sequence of spikes with high firing rate, separated by time windows of low or no firing. There is no exact mathematical definition of what constitutes a burst, and many different approaches for burst detection in a sequence of spikes have been proposed (e.g. [52], [72]–[75]). Furthermore, every approach needs to be slightly tuned to the parameters of the neurons under investigation, because different neurons may have slightly different background firing rates or refractory periods.

For our study we defined a heuristic set of rules to extract bursts from the recordings, which is similar to the method used in [72]. Before a burst starts, there must be a time window of at least 60 ms in which no spike occurs. The first spike of a burst must be followed by another spike no later than 15 ms afterward. The end of the burst is detected when the first time interval of 30 ms or longer occurs, or if two consecutive intervals combined are longer than 45 ms. A burst is only accepted as such if it contains at least 5 spikes and is longer than 8 ms. Figure 2D illustrates the criteria that define a burst.

This set of rules could reliably extract all bursts from the recordings, which are often obvious from visible inspection. A variant of this algorithm has been used for previous studies [21]. Other burst extraction methods did not lead to a (subjectively) better performance.

When artificial stimuli were broadcast to the animals, most stimuli were followed by a burst in the omega neuron with a short latency of about 10 ms. We associated stimulus and burst if the onset of the burst occurred not more than 50 ms before or after the onset of the stimulus (the burst may start before the stimulus if the burst detection algorithm includes spikes elicited by background noise immediately before the stimulus associated burst begins). Every burst was assigned one of 6 class labels: it is either associated with one of the five different stimuli (see Figure 1B), or it is a noise burst, in response to a random signal in the acoustic background.

### Spike Metrics

Spike train data typically comes in the form of sequences of firing times of variable length, which is not compatible with the requirements of traditional machine learning methods to receive inputs in the form of fixed-size, real-valued vectors. One can circumvent this problem by discretizing the spike trains into time bins, or extracting sets of numerical features, but both methods inevitably lead to a loss of information and temporal precision. Preserving temporal precision in spike timing is a necessary prerequisite for the analysis of auditory neural codes, where fast temporal fluctuations provide important information about the nature of the incoming stimulus. One way to analyze neural data without further preprocessing, is to use non-parametric machine learning methods (see e.g. [34], [35]), which only require a similarity measure between data points.

In order to find re-occurring burst patterns in neural signals, one therefore needs an objective measure of how similar two bursts are. Many different similarity measures [17], [64], [76], [77] and kernel functions [78], [79] for sequences of spikes have been proposed in the literature.

We chose to use the spike-time metric by Victor and Purpura [15], which computes the minimal costs of transforming one spike train, represented by the times of spikes, into the other. The algorithm applies three operators with variable costs for the transformations: Insertion or deletion of a spike at an arbitrary time point constitutes a cost of 1. The third operator is a shift of a single spike time, which has a cost of 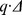, where 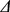 is the duration of the shift, and *q* is a parameter (with unit 1/ second) that needs to be defined in advance. High *q* will tend to prefer insertion and deletion of spikes to shifting, so small spike-time differences have a large influence on the distance. Low *q*, on the other hand, is more tolerant with respect to small spike-time differences, so the dominating factor is the difference in the number of spikes in the two bursts. Shifting is preferred to insertion and deletion as long as two spikes occur within an interval of 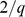 seconds [17].

Since the calculation of the distance matrix for a particular value of 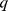 and subsequent clustering for a dataset with up to 20,000 bursts is very time consuming (up to 24 hours per recording), we had to determine the choice of the metric and a suitable value for 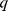 a-priori. For this experiment, we used one of the smaller datasets, which contains 3936 bursts. The decision to use the spike-time metric from [15] was based on results from a supervised classification experiment, in which we compared the performance of a *k*-Nearest Neighbor classifier (for *k* between 1 and 7) for different metrics and *q* values. The other metrics we tested were the van Rossum metric [64], and the Victor-Purpura interspike-interval distance [17], for various choices of the metric cost parameters.

**Figure 13:**
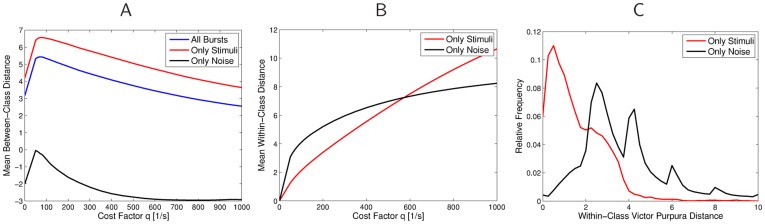
Analysis of the influence of the *q*-parameter for the Victor Purpura metric. The analysis was performed on a single dataset, consisting of 3936 bursts. **A**) Separability of different classes for different values of the parameter *q* for the Victor Purpura metric [15]. The plot shows the difference between the average between-class and within-class distances, separately plotted for the whole dataset (blue), for stimulus-classes only (red), and for noise only (black). The best separability is obtained for *q* values between 50 and 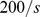. **B**) Average within-class distances for difference choices of *q* for bursts with identical spike count in response to artificial stimuli (red), or in response to noise (black). Our choice of 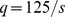 lies in a region of high similarity for the more stereotypical stimulus bursts, and low similarity of the irregular noise bursts, which is desirable. **C**) Histograms of spike time distances within the same class, for cost 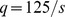, and for bursts that have the same number of spikes, either in response to artificial stimuli (red), or in response to noise (black).

For the final choice of the *q*-parameter of the spike-time metric we computed the differences between the average spike train distances of bursts of different classes, compared to the distances within the same class. A high difference indicates that the different classes can be well discriminated. Our analysis for *q*-values between 0 and 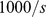 showed that bursts of different classes can be best discriminated using values of *q* between 50 and 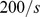 (see Figure 13A). After visual inspection of the clustering results for several values of *q* between 50 and 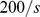, we identified 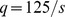 as the parameter which in general led to the best results, based on homogeneity of the clusters, the resulting number of clusters, as well as silhouette values [80], which are indicators of cluster qualities. We also found, that the same value of 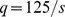 produces very good results for all recordings, but we did not attempt to optimize *q* for every single session.

The significance of using the parameter value 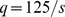 is that in the metric the cost for deleting or inserting a spike is the same as shifting a spike for 16 ms. This does not mean, however, that spike-time differences of less than 16 ms are neglected in the metric, but rather should be seen as a relative indicator of precision. In the February 22 dataset used for detailed analysis, we find an average spike timing distance of 12.2 between all bursts, in comparison to an average difference in the number of spikes of 7.8. Obviously, the spike-count difference is a lower bound for the Victor Purpura metric, and for bursts with an identical number of spikes, only the setting of *q* determines the range of values observed for the metric. For 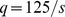, bursts that have the same number of spikes have an average distance of 4.98, which is mostly due to shifts. Bursts of identical length from the same class are even closer: two bursts from the same stimulus class have an average distance of 1.68, and the average distance between noise bursts is 4.43. In Figures 13B we show how the average distance of bursts with identical spike count and from the same class changes with *q*. The two curves show the average within-class distance for stimulus bursts (computed separately for all 5 classes, and then averaged), and for noise bursts. The more irregular class of noise bursts has a higher average within-class distance for *q* values below 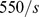. For higher *q*-values the noise bursts appear to be more similar on average than stimulus bursts, but at the same time, the variance of the distance values grows much stronger than for the stimulus bursts (not shown here). For decoding, one would like to have lower distances between the more stereotypical bursts in response to artificial stimuli and high distances between the irregular noise bursts, which is fulfilled by our choice of 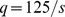. Figure 13C shows for 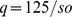 the distribution of distance values for bursts from the same class and with identical spike count. One can see that the within-class distances for stimulus bursts are lower and more concentrated than for noise bursts.

Although we made use of the supervised label information to determine a good value for *q*, it is important to note that this information is never used in the clustering, and that this does not violate our claim that bursts can be categorized based only on the similarity of their firing patterns. Although we do by no means claim that bushcrickets use the same metric, it is easily imaginable that a parameter like *q* could be tuned by evolution, since it only indicates how much deviation from an expected pattern can be tolerated in order to recognize a well-known stimulus. We view our results as a first step towards demonstrating the potential of unsupervised machine learning methods for the analysis of spike patterns in real-world spike train recordings, and do not claim that our results are “optimal”, especially since there is no unique criterion for judging the optimality of a clustering result.

One problem with the automatic detection of bursts is that spikes that are results of background noise, rather than responses to the stimulus that caused the burst, may occur shortly before the beginning of a burst. These spikes cannot be separated from the rest of the burst if they fulfill all criteria for a burst spike. For the metric proposed in [15], a burst that is temporally shifted because of noise spikes before the actual burst can look very different from another burst with the same temporal pattern, but without initial noise spikes (see Figure 6). We therefore modified the metric of [15] and introduced another operation, the *burst-shift* operator. The burst-shift operator can delete up to 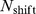 initial spikes from every burst for a cost of 1 per spike. It then re-aligns the two spike trains by shifting all spike times such that the new first spike occurs at time 0. The distance between the remaining spike trains is computed with the standard metric from [15], and added to the cost of deleting the initial spikes. This operation is different from deletion of single spikes, because it shifts all subsequent spike times within the burst by a constant value. This is sensible for our task, because we are not interested in absolute firing times, but only in firing patterns relative to the burst onset. For our purposes we set 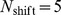. This compensates occasional unavoidable errors in the burst detection process.

For two spike trains *A* and *B* the distance 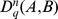 between the two spike trains is defined, using the cost factor *q* and 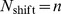 (for concise notation, *q* and *n* will be omitted wherever obvious). For two sets of spike trains 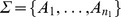 and 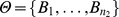, we define a 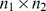 distance matrix 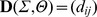, where 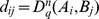. For a single set of bursts 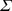 the matrix 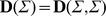 yields all distances between bursts within the same data set.

### Clustering with Affinity Propagation

Clustering is an unsupervised machine learning technique that finds groups of related data objects, based on a measure of similarity or distance. Many of the standard clustering methods, like e.g. *k*-means [81], are not suitable for clustering spike train data, because they work only in Euclidean space, and require direct manipulations of the data points, e.g. for computing means of groups of data points. For neural recordings it is not defined what the “average” spike train of a group of multiple spike trains is. Clustering a set of spike trains 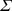 therefore requires a clustering algorithm that works only on the matrix of spike-train distances 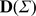, or equivalently the similarity matrix 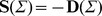. There are several clustering algorithms that meet this requirement, such as e.g. *k*-medoids, hierarchical clustering methods [81] or spectral clustering [82]. For this study the affinity propagation algorithm by Frey and Dueck [18] was chosen, which is very fast and reliable, and which has exhibited superior performance over comparable methods on a variety of datasets.

The affinity propagation algorithm uses only the information contained in the similarity matrix 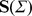, which we obtained from the spike-train distances. Affinity propagation defines every cluster through one central data point, the cluster *exemplar*. The algorithm assigns all data points to clusters in order to minimize an energy function based on the similarity between data points and their assigned exemplars. In contrast to previous clustering methods like *k*-medoids, all points are simultaneously considered as potential exemplars, instead of initially picking random data points as exemplars. A random choice of exemplars might lead to completely different results for every run with new initial random assignments. Instead, affinity propagation needs to be run only once, and will always come up with the same or very similar results. A further advantage is that the number of desired clusters does not have to be specified in advance. In contrast, the number of clusters as well as the cluster exemplars and the assignments of data points to exemplars emerge from an iterative message passing algorithm on a factor graph representation [83] of the data set.

Two different types of messages are used in this algorithm: The first one is a responsibility message 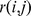, which is sent from a data point *i* to a potential exemplar *j*. This message represents the preference of data point *i* to choose *j* as its exemplar, relative to the preferences for all other potential exemplars. So 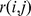 will be high, if *j* has clearly emerged as the exemplar for *i*, but low if there are many competing candidate exemplars. The other type of message is the availability message 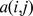, which is sent in the opposite direction from the potential exemplar *j* to the data point *i*. It indicates the evidence for point *j* becoming an exemplar, based on the responsibility of *j* for all other data points. This message will be high if many points have chosen *j* as exemplar. The responsibility and availability messages depend on each other, and so an iterative update of the messages is required to compute the clustering.

Initially, all 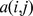 messages are set to zero. In every iteration the responsibilities 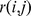 are computed based on the similarities 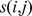 from 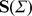 and the previous 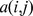. Then the availabilities 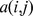, and self-availabilities 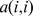 are updated from the new responsibility values. The update equations, described by Frey and Dueck in [18] are:
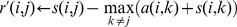
(1)

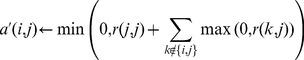
(2)

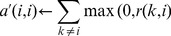
(3)


In our case this iterative procedure is run for a predefined number of steps (200 in all our experiments). Exemplars are then defined for every data point *i* by finding the data point 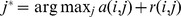, i.e. the exemplar needs to have high availability and a strong responsibility for *i*. If 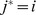, then *i* is its own exemplar.

We implemented the algorithm in Matlab, using the Distributed Computing Toolbox for processing large similarity matrices. Following the advice in [18], the updates for *r* and *a* were smoothed by a factor 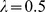, so that e.g. 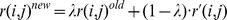. The rationale for this is to prevent numerical oscillations. We also added very small Gaussian noise (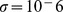 in all experiments) to the similarity matrix to avoid potentially ambiguous cluster assignments.

The number of clusters can be implicitly controlled by scaling the diagonal entries 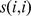 of the similarity matrix, which define the preference of a data point to choose itself as its exemplar. Larger self-preference values lead to a larger number of clusters. While normally the distance of a spike-train to itself is 0, a successful strategy in practice is to set the self-preference 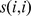 to the median of similarities 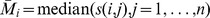, which leads to a moderate number of clusters. We employed this strategy, and in addition multiplied the self-preferences with constants 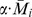 between 1 and 20. In our case the 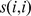 are negative distances, and so multiplication with 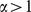 decreases the self-similarities and leads to a smaller number of clusters.

In contrast to other methods that require the exact number of desired clusters as input, this method is much easier to tune to obtain satisfactory results. The method is also insensitive to random assignments of cluster exemplars, so it is not necessary to run the clustering algorithm multiple times and choose the best clustering.

### Creating Cluster Dendrograms

To visualize the results of the clustering algorithm, the clusters are arranged in a dendrogram, applying the group average hierarchical clustering method [81] to the distances of the cluster exemplars. The algorithm starts by assigning every cluster to a single unconnected branch, and then at every step joins the two closest branches into a new higher level branch. Closeness between branches is defined as the average spike-time distance between all cluster exemplars contained in the two branches. As a result, clusters with similar exemplars are grouped together in the same branch. This procedure is applied recursively until all branches have been joined to form a single tree.

### Labeling of Clusters

Cluster labels are assigned based on the relative frequencies of burst classes within the cluster and in the whole dataset. For a cluster 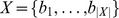 consisting of bursts 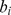, we define 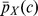 as the relative frequency of bursts of class *c* in *X*. We also define 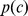 as the relative frequency of class *c* in the whole dataset. The cluster label 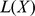 is the class *c* with the highest ratio 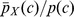.

### Measuring Cluster Homogeneity

As a measure of cluster homogeneity we used the average conditional entropy of class labels for every cluster. The conditional entropy 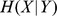 is an information-theoretic measure, which quantifies for two random variables *X* and *Y* (here assumed discrete), the uncertainty about *X* given that *Y* is known. Formally, the conditional entropy is defined as the difference between the joint entropy 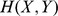 and 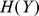, the entropy of *Y* alone. It is calculated as
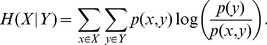
(4)


The conditional entropy is low if knowing variable *Y* reduces the uncertainty about *X*, and reaches its highest possible value 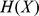 when *X* and *Y* are independent. In our case this would mean that knowing the cluster label should reduce the uncertainty about the classes of bursts that are found within the cluster, so in the ideal case there should be only one class of bursts in every cluster (which means zero entropy). For every stimulus class *c* we measure the conditional entropy of variable 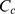, which indicates whether a burst *b* belongs to class *c* (in that case 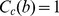), or to another class (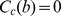), relative to variable *L*, which indicates the identity of the cluster (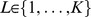, where *K* is the number of clusters). Only clusters that contain at least one burst of class *c* are considered for computing 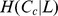. For each of the six recording sessions we compute the clustering, and measure the conditional entropy 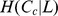 separately for every stimulus class 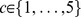.

### Ethics Statement

All necessary permits were obtained for the described field studies. The Smithsonian Tropical Research Institute granted access to protected rainforest area for insect research to Prof. Heinrich Römer. The experiments reported in this paper comply with the current animal protection law in Austria, and with current Panamanian laws. According to these laws, studies on insects do not require approval by a review board institution or ethics committee. The study was conducted on Barro Colorado Island (BCI; 009’N, 7951’W), Panama, in February/March and June/July 2002,2003, and 2004, in the dry season and at the beginning of the rainy season, respectively. We studied *Docidocercus gigliotosi*, a pseudophylline katydid which is one of the most common katydids on the island.
